# Simultaneous 18-FDG PET and MR imaging in lower extremity arterial disease

**DOI:** 10.3389/fcvm.2024.1352696

**Published:** 2024-02-09

**Authors:** Tobias Koppara, Isabel Dregely, Stephan G. Nekolla, Jörg Nährig, Nicolas Langwieser, Christian Bradaric, Carl Ganter, Karl-Ludwig Laugwitz, Markus Schwaiger, Tareq Ibrahim

**Affiliations:** ^1^ Department of Internal Medicine I, Cardiology and Angiology, School of Medicine and Health, Technical University of Munich, Munich, Germany; ^2^ DZHK (German Center for Cardiovascular Research)—Partner Site Munich Heart Alliance, Munich, Germany; ^3^ Department of Nuclear Medicine, School of Medicine and Health, Technical University of Munich, Munich, Germany; ^4^ Institute of Pathology, School of Medicine and Health, Technical University of Munich, Munich, Germany; ^5^ Institute of Radiology, School of Medicine and Health, Technical University of Munich, Munich, Germany

**Keywords:** optical coherence tomography, magnetic resonance imaging (MRI), FDG PET = F-18 fluorodeoxyglucose positron emission tomography, atherectomy, peripheral arterial disease

## Abstract

**Background:**

Simultaneous positron emission tomography (PET) and magnetic resonance imaging (MRI) is a novel hybrid imaging method integrating the advances of morphological tissue characterization of MRI with the pathophysiological insights of PET applications.

**Aim:**

This study evaluated the use of simultaneous 18-FDG PET/MR imaging for characterizing atherosclerotic lesions in lower extremity arterial disease (LEAD).

**Methods:**

Eight patients with symptomatic stenoses of the superficial femoral artery (SFA) under simultaneous acquisition of 18-FDG PET and contrast-enhanced MRI using an integrated whole-body PET/MRI scanner. Invasive plaque characterization of the SFA was performed by intravascular imaging using optical coherence tomography. Histological analysis of plaque specimens was performed after directional atherectomy.

**Results:**

MRI showed contrast enhancement at the site of arterial stenosis, as assessed on T2-w and T1-w images, compared to a control area of the contralateral SFA (0.38 ± 0.15 cm vs. 0.23 ± 0.11 cm; 1.77 ± 0.19 vs. 1.57 ± 0.15; *p*-value <0.05). On PET imaging, uptake of 18F-FDG (target-to-background ratio TBR > 1) at the level of symptomatic stenosis was observed in all but one patient. Contrast medium-induced MR signal enhancement was detected in all plaques, whereas FDG uptake in PET imaging was increased in lesions with active fibroatheroma and reduced in fibrocalcified lesions.

**Conclusion:**

In this multimodal imaging study, we report the feasibility and challenges of simultaneous PET/MR imaging of LEAD, which might offer new perspectives for risk estimation.

## Introduction

Atherosclerotic disease is the leading cause of mortality and morbidity in the Western world and an increasing burden to healthcare systems worldwide (1). Over the years, plaque formation in the arterial vessel wall may occur clinically silent but may also present with an acute rupture of plaque material in vulnerable lesions, resulting in acute coronary, cerebral, and peripheral arterial complications. The presence of atherosclerotic lesions in one arterial territory is a strong predictor of plaque rupture and thrombosis in other territories (2). Lower extremity arterial disease (LEAD) is a common yet under-diagnosed manifestation of atherosclerosis associated with significant morbidity (3). The vulnerability of an atherosclerotic plaque is determined by its histological composition. Inflammation plays a key role in the initiation and progression of atherosclerosis and is closely related to plaque vulnerability (4). Consequently, non-invasive imaging modalities yielding information about plaque constituents may improve risk estimation of adverse events in LEAD, such as acute limb ischemia.

Positron emission tomography (PET) using 18-fluorine-fluorodeoxyglucose (18F-FDG) has been shown to assess inflammation in atherosclerosis within different vascular regions, including the aorta, carotid, iliac, femoral, and coronary arteries (5, 6). FDG uptake measured within atherosclerotic carotid plaques is closely correlated with the macrophage density (7). However, although PET imaging allows excellent spatial coverage, it is limited by a low spatial resolution (approximately 4 mm). In contrast, magnetic resonance imaging (MRI) of plaques has the advantage of high spatial resolution and provides information on both plaque volume and composition in multiple arterial territories (8). Several studies of the carotid artery and aorta have demonstrated that different plaque components, such as fibrous cap, lipid-rich necrotic core, hemorrhage, and calcification, can be differentiated by employing multiple contrast weightings (9–11). In addition, contrast-enhanced MRI using gadolinium (Gd)-based contrast agents provides enhanced visualization of the fibrous cap and has been shown to reveal plaque vascularity and inflammation in dynamic imaging studies (12–14).

Consequently, multimodal imaging techniques simultaneously combining PET using fully integrated, whole-body PET/MR systems may offer new opportunities for the non-invasive characterization of atherosclerosis. The aim of this pilot study was threefold: to assess (1) the feasibility of LEAD imaging using an integrated, whole-body PET/MR system, (2) the relationship between FDG PET and MR contrast agent uptake in LEAD, and (3) the potential of non-invasive plaque characterization by comparing non-invasive PET/MR imaging to intravascular imaging by optical coherence tomography, an imaging modality that has proven high-resolution imaging of LEAD plaques, and histology of plaque specimen.

## Methods

### Patient population

The study protocol was reviewed and approved by the local ethics committee, and written informed consent was obtained prior to inclusion. Ten patients with LEAD and intermittent claudication (Rutherford categories 2 and 3) were recruited in this study. Patients were considered eligible if they had one or more symptomatic *de novo* atherosclerotic lesions with at least 50% stenosis in the superficial femoral artery, as assessed by duplex ultrasound, and if they had been referred for peripheral artery atherectomy. All patients underwent clinical examination and physiological testing at the Department of Angiology of our institution, which included assessment of the ankle–brachial index (ABI) and color flow duplex sonography (Siemens Acuson S2000, Erlangen, Germany) of the lower limbs.

Duplex criteria subjected to predict hemodynamically relevant stenosis included a maximum peak systolic velocity >200 cm/s, loss of triphasic waveform configuration at the stenosis, damping and reduction in pulsatility in the distal arterial velocity waveform, a peak-systolic (PSV) and end-diastolic (EDV) velocity ratio >2.5 across the stenosis, and end-diastolic velocity >60 cm/s. The lesions included were regarded as appropriate for treatment by atherectomy if they were classified as TASC A or B lesions according to the TASC II classification by two independent experienced physicians (15).

Patients were excluded if they had critical limb ischemia, total occlusion of the target superficial femoral artery, severe renal insufficiency, type 1 diabetes mellitus, or contraindications to PET and/or MR imaging. All patients underwent PET/MR imaging within 24 h prior to atherectomy of the symptomatic lesions.

### Imaging protocol

Patients fasted for at least 12 h before the intravenous injection of 18F-FDG and simultaneous PET/MR imaging in a whole-body scanner (Biograph mMR, Siemens Erlangen, Germany). An attachable marker was placed on the skin to indicate the area of the stenotic vascular region of interest, as assessed by color flow duplex sonography to indicate the plaque location for PET/MR imaging and atherectomy. For PET imaging, a 20-min scan was acquired at a single bed position (longitudinal FOV = 25 cm), centered at the position of the attached marker. Before each PET acquisition, an 18-s MR Dixon sequence was acquired to generate a fat-water separated map for attenuation correction (AC) of PET data (16). PET imaging parameters were as follows: 3D mode, 172 × 172 matrix, 4.17 × 4.17 mm pixel spacing, 2 mm slice thickness, and 127 slices. Images were reconstructed using the ordered-subsets expectation maximization (OP-OSEM) algorithm with a 4-mm Gaussian filter. Simultaneously, MR imaging was performed using a combination of body matrix and spine array coils to cover both upper legs. The following MRI sequences were acquired: time-of-flight (TOF), T1-w turbo spin echo (TSE), and T2-w TSE. Detailed imaging parameters for MRI sequences are listed in Supplementary Table S1. The T1-w sequence was repeated 5–7 min after injecting the Gd-based contrast agent (Magnevist, Bayer Schering Pharma AG, Berlin, Germany) at a dosage of 0.1 mmol/kg body weight). The total imaging time averaged 45–60 min.

### Image analysis

Image analysis was performed using MATLAB (MathWorks, Natick, MA, USA). On postcontrast T1-w axial MRI images, the plaque size (maximum diameter) was measured. PET and MRI images were fused, and the location of the plaque was determined by selecting the postcontrast T1-w MRI image slice with the largest plaque (“target” slice). For MRI image analysis, a ring-shaped region of interest (ROI) was drawn around the arterial vessel wall, excluding the luminal signal, i.e., blood. A circular ROI in the adjacent musculus quadriceps femoris was used for normalization. The MR plaque enhancement ratio (ER) was calculated as previously described (17).

For PET analysis, FDG uptake was assessed on the axial image identified by MR (“target”). The ring-shaped ROI delineated on MRI images was copied to the fused PET image. Due to the low resolution of PET, the enclosed circular ROI was used (including lumen) for the artery signal. For further analysis, the ROI maximum standard uptake value (SUV_max_) was used as previously described (18). The arterial PET signal was normalized to the average standard uptake value (SUV_average_) in the adjacent muscle ROI. This yielded the target-to-background ratio (TBR):

$$\mathrm{TBR}=\frac{{\left(\mathrm{SU}V_{max}\right)}_{\mathrm{artery}}}{{\left(\mathrm{SU}V_{max}\right)}_{\mathrm{muscle}}}$$

The analysis was also performed for the contralateral (asymptomatic) superficial femoral artery. The overall intensity of FDG uptake in femoral plaques was measured by max. SUV and the tissue-to-background ratio (ratio of max. SUV of plaque/mean SUV of blood) on PET and compared among calcified and non-calcified plaques.

#### OCT image acquisition

Intravascular imaging was performed using angiography and a frequency-domain OCT system (Ilumien OCT-Imaging System, Abbott Vascular, Santa Clara, CA). A 6-French guiding catheter was placed proximal to the stenotic arterial segment to provide sufficient flushing. An OCT catheter (Dragonfly Imaging Catheter, Abbott Vascular, Santa Clara, CA, USA) was inserted distal to the SFA target segment after the passage of a 0.014-in guide wire. The entire length of the stenotic region was scanned using an automated OCT pullback at a speed of 20 mm/s. For image acquisition, flushing was obtained using iso-osmolar contrast media at a rate of 5–8 mL/s, allowing sufficient blood clearance. Intravascular imaging using OCT was performed in addition to the routine angioplasty procedures, and it did not affect decision-making regarding the treatment of the individual patient.

#### OCT image analysis

The target segment for OCT analysis was defined as the SFA segment exhibiting the minimal luminal diameter as assessed by angiography and the adjacent vascular segment ranging at least 20 mm proximally and distally. Raw data from OCT pullbacks were collected and analyzed offline. Each OCT sequence was assessed and analyzed by independent investigators, and a decision was based on consensus. All cross-sectional images (frames) were screened for image quality, and those with <270° of the cross-sectional image visible and limited by residual blood or thrombus were excluded from the analysis. According to previous reports, thrombus was defined as a mass attached to the luminal surface or floating within the lumen with either high backscattering, heterogeneous appearance, and high attenuation (red blood cell-rich thrombus) or low backscattering, homogeneous appearance, and low attenuation (white platelet-rich thrombus) (19).

#### OCT plaque characterization

As previously defined, fibroatheromas were characterized by a signal-poor region within an atherosclerotic plaque, exhibiting high attenuation with diffuse borders and little or no backscattering (20). For fibroatheromas, a lateral plaque extension of at least one quadrant was required. Fibrocalcific plaques comprised a signal-poor or heterogeneous region with low attenuation and clearly visible demarcations of its borders. A fibrous plaque is characterized by high backscattering and a relatively homogeneous OCT signal.

### Tissue collection and histology

All patients underwent angiography of the target lesion via an antegrade percutaneous femoral artery access using a 7-F sheeth within 24 h after PET/MR imaging. The selected symptomatic lesions of the superficial femoral artery were treated with a SilverHawk plaque excision system (Covidien ev3 Endovascular, Inc., Plymouth, MN, USA) by an experienced interventionist. The number of lesion passes and the use of an additional low-pressure balloon were left to the discretion of the interventionist.

Immediately after excision, specimens obtained by atherectomy were fixed using neutral-buffered formalin. After fixation, specimens were processed for histopathology. Decalcification was carried out after the specimens have been thoroughly fixed and prior to embedding in paraffin using ethylenediaminetetracetic acid (EDTA) at a concentration of approximately 14% as a neutralized solution. All portions were sectioned at 3–5 µm thickness. Sections were stained using hematoxylin and eosin (H&E) and Elastica van Gieson (EVG) staining methods. Immunohistochemistry included staining with antibodies targeting monocytes (CD-68) and alpha smooth muscle actin (SMA). Computerized planimetry of the stained area was performed using Image Pro Plus 6.1 (Media Cybernetics, Inc., USA) on all sections, as previously described (21). The histology slides were reviewed independently by a reviewer (J.N.) who was blinded to the imaging results and were categorized according to the AHA classification based on the histopathological classification described by Stary et al. (22, 23). A summary of advanced atherosclerotic lesions and the conventional AHA histological classification is provided in Supplementary Table S2. In this classification, the term *advanced lesion* is used for all lesions that disrupt intimal structure. Type V and VI lesions develop and progress by mechanisms that are superimposed on the continuing lipid accumulation that produced lesion types I–IV. In type IV lesions, the intimal structure is altered almost solely by an extensive accumulation of extracellular lipids localized in the deep intima (the lipid core). In type V lesions, the intima is thickened by substantial reparative fibrous (mainly collagenous) tissue layers (fibroatheroma). Type VI lesions are characterized by surface defects, hematoma, and thrombotic deposits. The predominant (often sheath-like) calcification of a fibro-lipid lesion corresponds to type VII (calcific lesion), while fibrous tissue layers without or with only minimal lipid accumulation (no core) and minimal or no calcium are described as type VIII (fibrotic lesion) (22, 23).

### Statistical analysis

All data were expressed as mean ± standard deviation. For the comparison of MR and PET signal uptake in the left vs. right superficial femoral artery, data were pooled and assessed using the Wilcoxon rank-sum test. Correlations between imaging parameters were assessed using Spearman's correlation coefficient testing. For all analyses, *p* < 0.05 was considered significant.

## Results

### Patient population

Ten patients were enrolled in this pilot study. Two patients were excluded from the data analysis because of insufficient imaging quality. A total of eight patients were included in the data analysis. The study population had a mean age of 70 ± 6 years and consisted of 75% male patients. Patients exhibited typical cardiovascular risk factors, as shown in Table 1. The majority of patients (88%) exhibited atherosclerosis in other vascular segments, including the peripheral and coronary vasculature. Three patients (38%) had a history of prior stroke or carotid artery disease. All patients were receiving antiplatelet and statin therapy. The mean ankle-brachial index (ABI) on the symptomatic side was reduced, averaging 0.8 ± 0.07. The mean maximum peak velocity within the stenosis averaged 313 ± 92 cm/s.

**Table 1:** Patient characteristics.

Patient characteristics	
Risk factors	
Hyperlipidemia (*n*, %)	*n* = 8 (100%)
Diabetes (*n*, %)	*n* = 5 (62.5%)
Hypertension (*n*, %)	*n* = 8 (100%)
BMI (kg/m^2^)	25.6 ± 2.5
Age (mean ± SD)	68.5 ± 8.2
Male (*n*, %)	*n* = 7 (87.5%)
Smoking (*n*, %)	*n* = 8 (75%)
Known LEAD (*n*, %)	*n* = 7 (87.5%)
ABI (mean ± SD)	0.85 ± 0.07
Known atherosclerosis of other vascular regions (*n*, %)	*n* = 7 (87.5%)
Cerebral/carotid (*n*, %)	*n* = 3 (37.5%)
Coronary (*n*, %)	*n* = 7 (87.5%)
Medication	
ASS (*n*, %)	*n* = 8 (100%)
Statin therapy (*n*, %)	*n* = 8 (100%)
Laboratory findings	
TC (mg/dL)	185.1 ± 42.3
LDL-C (mg/dL)	107.3 ± 36.0
Hs-CRP (mg/dL)	1.4 ± 3.1
TG (mg/dL)	247.5 ± 323.1

Values are mean ± SD, *n* (%). Hs-CRP, high-sensitivity C-reactive protein; LDL-C, low-density lipoprotein cholesterol; TC, total cholesterol; TG, triglyceride.

### Magnetic resonance imaging

**Figure 1:**
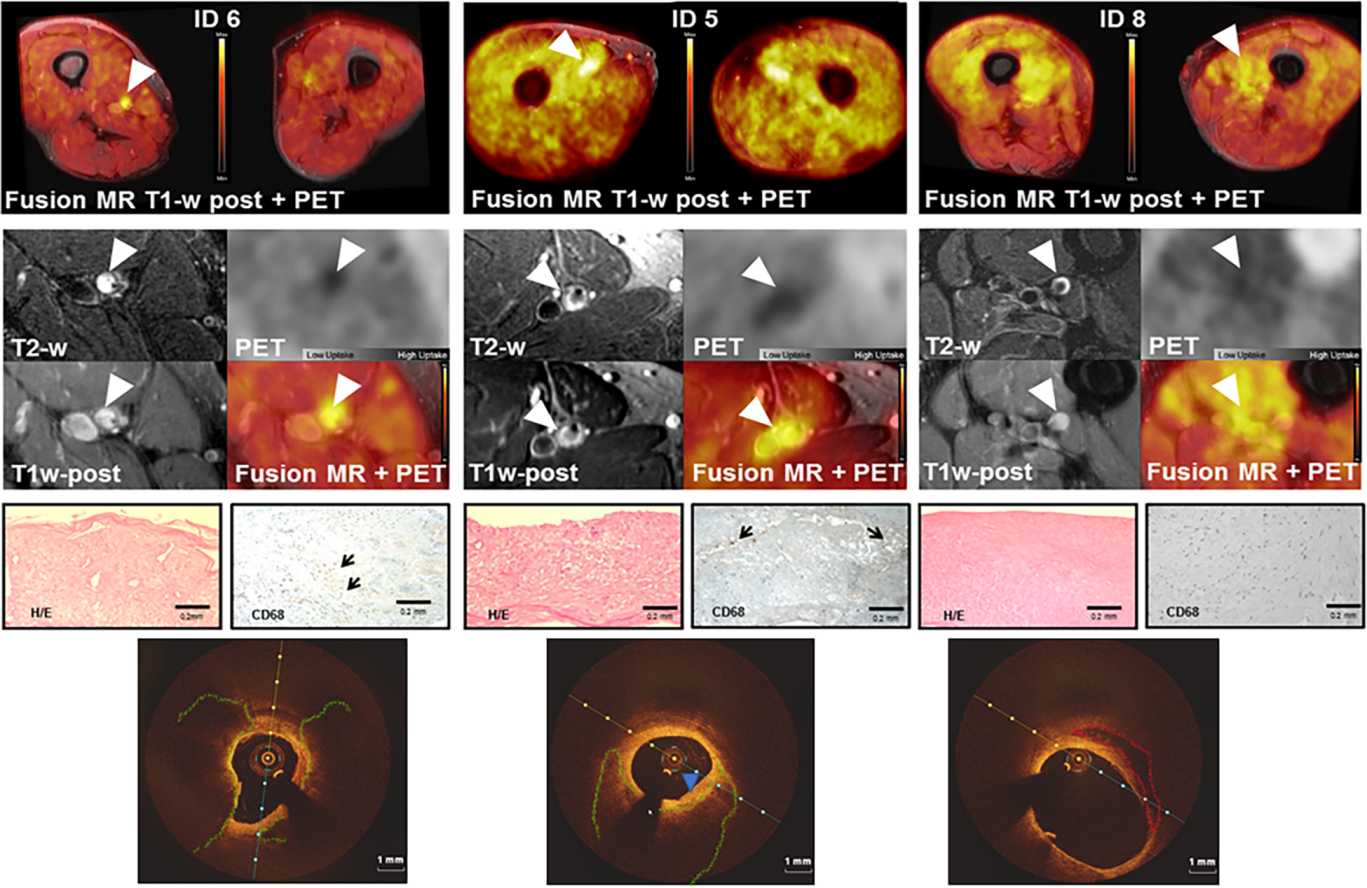
Representative coregistered images of PET/MR imaging and corresponding histology and optical coherence tomography. The upper part of the figure shows representative images of target stenotic lesion (arrowhead) in patients with different uptake patterns of 18F-FDG along the superficial femoral artery in simultaneous PET/MR imaging. The mid part of the figure shows zoomed axial view MR images of the target stenotic lesion (arrowhead) detailing plaque characterized by positive remodeling on T2-w MR. The plaque shows Gd-based contrast agent enhancement on T1-w post-contrast images of MR. MR and PET images are inherently coregistered and fused due to the simultaneous acquisition. In the lower part of the figure, plaque histology shows fibrous plaques and interspersed inflammatory cells as depicted by CD68 positive macrophages (arrow) and corresponding OCT frames showing the target stenotic area of the SFA. Signal-poor fibrous plaques with diffuse borders and little or no backscattering are delineated in green, whereas fibro-calcific plaques are shown in red. CD68 = antibody to CD68 (24).

**Figure 2:**
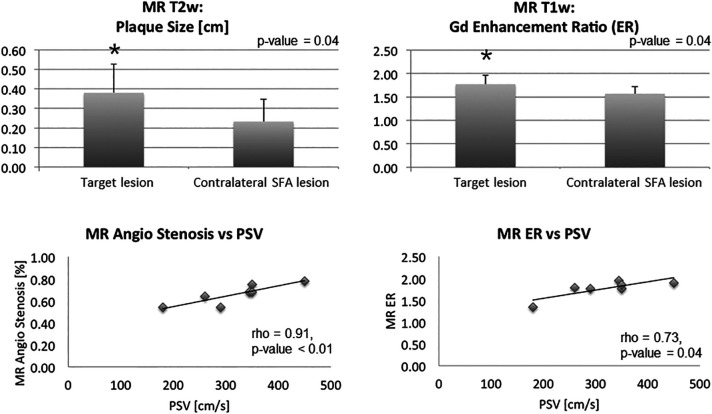
MR imaging results. (**A**) MR plaque size measurements based on T2-w axial images showed a significant larger plaque size in the diseased target lesion compared to a contralateral SFA lesion. (**B**) MR contrast agent enhancement was higher in the diseased leg than in a contralateral SFA lesion on T1-w axial images. (**C,D**) Correlation of MR imaging parameters showed a significant correlation of PSV with the degree of stenosis and PSV with MR contrast enhancement in the plaque.

Figure 1 shows the representative images of the multimodal imaging procedures performed on all study patients. Based on T2-w images, the measurements of plaque extent within the stenotic lesion of the symptomatic superficial femoral artery averaged a mean of 0.38 ± 0.15 cm, which was significantly larger compared to the contralateral control site (mean 0.23 ± 0.11 cm, *p*-value = 0.04), thus emphasizing positive remodeling of the target lesion (Table 2, Figure 2A). Similarly, MR contrast uptake at the level of the symptomatic stenosis assessed on T1-w images was significantly higher compared to the contralateral superficial femoral artery control area (mean 1.77 ± 0.19 vs. 1.57 ± 0.15; *p*-value = 0.04) (Table 2, Figure 2B). The mean degree of stenosis on MR-TOF angiography in the entire study population averaged 68 ± 11% and significantly correlated with the peak systolic velocity, as assessed by duplex sonography (*ρ* = 0.91, *p*-value < 0.01) (Table 2, Figure 2C). Contrast enhancement on T1-w imaging significantly correlated with the peak systolic velocity measurements by duplex sonography (*ρ* = 0.73, *p*-value = 0.04) (Table 2, Figure 2D).

**Table 2:** Imaging results.

Patient ID	1	2	3	4	5	6	7	8	Mean
Duplex sonography									** **
PSV (cm/s)	180	260	345	290	350	350	450	470	337 ± 95
MR									
Stenosis (%)	0.54	0.64	0.68	0.54	0.68	0.75	0.78	0.84	0.68 ± 0.11
T2-w: plaque size (cm)—diseased leg	0.51	0.15	0.28	0.26	0.47	0.44	0.34	0.59	0.38 ± 0.15
T2-w: plaque size (cm)—contralateral leg	0.31	0.15	0.11	0.1	0.27	0.26	0.44	0.22	0.23 ± 0.11
T1-w: Gd enhancement—diseased leg	1.34	1.79	1.94	1.77	1.76	1.85	1.90	1.82	1.77 ± 0.19
T1-w: Gd enhancement—contralateral leg	1.39	1.83	1.67	1.40	1.70	1.56	1.53	1.49	1.57 ± 0.15
18F-FDG PET									
Target-to-background (muscle) ratio	1.59	0.37	2.23	2.62	2.28	2.19	2.91	1.88	2.01 ± 0.78
(TBR)									
Tissue-to-background (blood) ratio (TBR)	0.99	0.86	1.50	1.40	0.88	1.51	0.92	0.89	1.12 ± 0.30
Histopathology									
Plaque type (AHA)	7	7	8	6	5	6	8	7	
CD68 (% area)	1.81	0.05	0.50	0.77	2.92	0.87	0.26	0.06	0.91 ± 0.99
SMA (% area)	8.70	4.61	2.60	0.01	0.04	2.81	7.24	9.64	4.46 ± 3.74
Optical coherence tomography									
Plaque type	Fibro-calcific	Fibro-calcific	Fibrous	Fibroatheroma	Fibroatheroma	Fibroatheroma	Fibrous	Fibro-calcific	
Sheath-like calcification	Yes	Yes	No	No	No	No	No	Yes	

Imaging results derived from duplex ultrasound, simultaneous MRI and 18-FDG-PET, histopathology, and optical coherence tomography. AHA, American Heart Association; PSV, peak systolic velocity; TBR, target-to-background ratio corrected for muscle and blood background signal; CD68, antibody to CD68; SMA, small muscle actin.

### Positron emission tomography imaging

**Figure 3:**
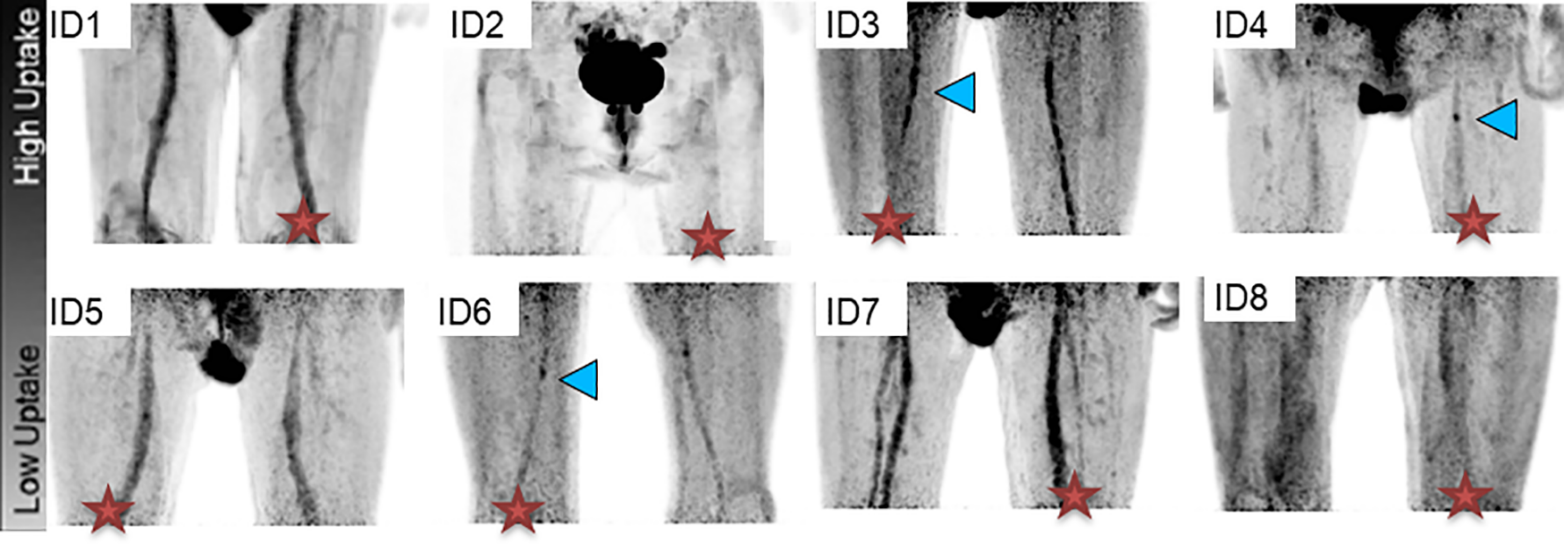
PET imaging results. Coronal views of 18F-FDG PET maximum intensity projections of the entire study population demonstrated different FDG uptake patterns among patients. A cross indicates the symptomatic side. Quantitative analysis [target-to-background ratio normalized to the muscle ROI signal (TBRm)] showed uptake (TBRm > 1) for all patients, except for patient ID 2.

Patients received a mean intravenous injection of 358 ± 33 MBq of 18F-FDG. After a mean circulation time of 2 h and 16 min ± 37 min, patients underwent simultaneous PET/MR imaging in a whole-body scanner (Biograph mMR, Siemens Erlangen, Germany) as shown in Figure 3.

Throughout the study population, different patterns of vascular 18F-FDG uptake were observed based on a qualitative assessment. In detail, one patient (ID 2) showed no tracer uptake. Four patients (IDs 3, 4, 6, 8) demonstrated focal FDG uptake of the symptomatic lesion, while in another three patients (IDs 1, 5, 7), tracer uptake was identified in the symptomatic lesion and the ipsilateral course of the superficial femoral artery.

Quantitative analysis showed the FDG-PET signal was enhanced in the symptomatic lesion compared to the blood signal only in the three patients with focal uptake (TBR > 1, Table 2). When comparing signal uptake in calcified vs. non-calcified plaques, we found no significant difference in FDG uptake (*p*-value = 0.40).

### Simultaneous PET/MR imaging

Simultaneous PET/MR imaging was successfully performed in all patients, allowing detailed postprocessing analysis of all modalities. Coregistered axial views of the atherosclerotic lesion on MRI allowed the integration of several plaque features such as positive remodeling, hyperintense signal on T2-w, and MR contrast agent uptake on T1-w images as well as 18F-FDG uptake. Representative images of simultaneous PET and MRI are shown in Figure 1. Assessing the relationship between PET and MR imaging parameters at the level of stenosis, we found no significant correlation between the PET-FDG TBR and the MR T1-w postcontrast agent enhancement (*ρ* = 0.29, *p*-value = 0.50).

#### Optical coherence tomography

The most frequent findings were fibroatheromas with thick fibrous caps (*n* = 5). Fibro-calcific plaques with clearly delineated sheet calcium were observed in three lesions. One lesion showed adjunct thrombus formation. Two lesions revealed signal-rich regions with high backscattering, likely representing macrophage infiltration. Representative OCT images are shown in Figure 1. Comparing histopathology with OCT imaging showed a trend toward increased TBR in fibroatheromas with low calcification and decreased TBR in fibro-calcified lesions (*p*-value = 0.14). Intraobserver and interobserver variability for neointimal tissue characterization was assessed in 30 randomly selected frames and showed high concordance (*κ* = 0.93 and *κ* = 0.88).

### Histopathology

All plaque specimens obtained by atherectomy consisted of fragments of fibrotic plaques and different extents of fibro-calcified components with interspersed macrophage infiltration. Histologically, we did not observe characteristic constituents of necrotic cores such as cholesterol crystals or intraplaque hemorrhage. Comparing histopathology with FDG-PET imaging showed no correlation but a weak trend toward increased TBR in fibrous plaques with low calcification and decreased TBR in fibro-calcified lesions (*ρ* = −0.25, *p*-value = 0.54). Interestingly, the patient with no 18F-FDG uptake (ID 2) had a late-stage fibro-calcified plaque with little to no macrophage deposition. Comparing histopathology with MR contrast enhancement showed no correlation. Staining for CD68 showed increased CD68-positve macrophages in plaques with no to little calcification, whereas it subsequently decreased within more fibro-calcified lesions, yielding a negative correlation (*ρ* = −0.73, *p*-value = 0.05). Comparison of CD68-staining positive macrophages with MR enhancement ratio showed a positive trend (*ρ* = −0.50, *p*-value = 0.22), whereas rather no correlation with MR plaque size measurements (*ρ* = 0.38, *p*-value = 0.36) or PET TBR (*ρ* = 0.24, *p*-value = 0.58). SMA within the plaque samples was rather low, not showing any correlation with imaging parameters.

## Discussion

In this observational pilot study, we assessed the feasibility and challenges of simultaneous PET and MRI in patients with symptomatic LEAD.

Hybrid PET/MR imaging combines the advantage of high-resolution MR with the molecular imaging sensitivity of PET. Axial high-resolution MR allowed for morphological characterization of the plaque, including the quantitative extent of positive remodeling and the delineation of plaque constituents. In our patient population, atherosclerotic plaques were predominantly characterized by hyperintense signals on T2-w images and retention of the Gd-based contrast agent. Although Gd-based contrast media are non-specific, this enhancement pattern is typically associated with mainly fibrotic plaques in LEAD, consistent with our OCT and histological findings of fibrotic and fibro-calcified plaques. Similar MR patterns have also been described in atherosclerosis within other vascular territories, such as the carotids and coronaries (10, 25). As derived from MRI and histology studies in patients with advanced carotid atherosclerosis, contrast enhancement varies in different plaque components and is particularly strong in fibrous plaques, whereas it is found to be reduced in plaques with large necrotic cores (12, 26, 27).. However, other main components of atherosclerotic plaques, such as the extracellular matrix, lipids, and particularly inflammation caused by macrophages, may potentially contribute to the contrast uptake.

18-FDG has been shown to accumulate in macrophages and thus offers the opportunity to identify and track monocyte-rich inflammation within the arterial vessel wall, as assessed by PET imaging (5, 27, 18, 28). However, 18-FDG uptake is unspecific, and the variability in the composition of atherosclerotic plaques among different vessel territories in humans can result in heterogeneous distribution patterns and imaging findings in PET/CT studies (18). Simultaneous acquisition of PET, when added to established imaging modalities, may thereby offer valuable complementary information to improve the assessment of atherosclerotic plaque composition in carotid, coronary, and peripheral artery disease (5, 29, 30). However, as previously reported, we observed no correlation of 18-FDG PET tracer uptake with cellular inflammation in SFA plaques (31), but we noted a trend toward increased uptake in fibroatheromas as compared to fibro-calcific plaques as identified by OCT. Other PET tracers might offer better characterization of LEAD plaques, but they were not available for human application at the time of initiation of our study. Gallium-68 DOTA-0-Tyr3-Octreotate (68Ga-DOTATATE) PET has shown potential in identifying vulnerable atherosclerotic plaques with high somatostatin receptor expression, while 11-carbon (11C)-choline and 18F-fluoromethylcholine (FMCH) tracers may improve the characterization of high-risk atherosclerotic plaques by detecting increased choline metabolism (32). These radiotracers might help to better characterize the pathogenesis of LEAD plaques and stratify the risk for adverse vascular limb events.

There are considerable technical challenges to overcome for simultaneous contrast-enhanced PET and MR imaging of atherosclerotic plaques. Aizaz et al. have discussed potential solutions for coronary and carotid artery hybrid PET/MR imaging, and we have previously reported our initial observations of PET/MR imaging in LEAD (31, 33). The inherent coregistration of simultaneously acquired PET and MRI images in our study facilitated ROI-based quantitative PET analysis. Interestingly, our patients showed varying vascular 18-FDG uptake patterns on 3D PET views, including no distinct 18-FDG uptake, clearly visible whole-vessel vascular uptake with additional focal uptake at the MRI-defined plaque location of the symptomatic SFA lesion, and whole-vessel vascular uptake without focal uptake at the MRI-defined plaque location. This potentially reflects the variable features and the global manifestation of atherosclerosis in our patients. It has been described that 18-FDG uptake may be increased in lipid-rich plaques, whereas plaques mainly consisting of collagen and those containing increased calcified areas showed lower 18-FDG uptake, thus suggesting a smaller number of active inflammation and macrophages in LEAD plaques as compared to carotid or coronary plaques (30). Our findings of increased target and contralateral femoral uptake, which we observed in three patients in this study, were similarly shown in a previous PET study by Myers et al. (28), likely representing a bilateral manifestation of LEAD. Extended vessel wall uptake might be an indicator of the presence of systemic inflammation in cardiovascular disease, as it has been shown in a population of cancer patients (34). Determining which plaques are susceptible to ischemia is essential for stratifying event risk. Unlike traditional risk assessments, PET radiotracers have the potential to visualize atherosclerosis-associated metabolic activity, identifying vulnerable plaques beyond morphologic characterization by MRI. Deep learning algorithms might offer promising solutions for improving imaging techniques and interpreting simultaneous PET/MRI findings in atherosclerosis (33).

In summary, we observed no correlation between 18-FDG PET and MR contrast enhancement imaging in our study cohort. We found no correlation between CD68 staining for macrophages and 18-FDG uptake, but we observed a trend toward increased uptake in fibroatheromas compared to fibro-calcific plaques identified by OCT. While numerous studies in the carotids found a correlation between 18-FDG uptake and CD68 staining (5, 35), Myers et al. also found no such correlation in LEAD (28). The authors hypothesized that the overall lower target-to-background ratio in LEAD compared to carotids might be associated with a lower degree of active inflammation in LEAD compared to carotid atherosclerosis (28, 36), which is corroborated by our findings [overall lower TBR in PAD (Myers et al.) = 1–1.5 (28), our study mean TBR = 1.12, Rudd et al. mean TBR∼1.4 (36) as compared to carotids]. As the lesions identified using intravascular optical coherence tomography all represented thick cap fibrous plaques or fibro-calcific plaques, vulnerable plaque stages, i.e., thin cap fibroatheroma or ruptured plaques, could not be imaged in our study.

Our study has several limitations. First, only a small number of patients were examined in this pilot study, thus prohibiting sound statistical conclusions. Statistically non-significant trends for a correlation between 18-FDG PET and MR contrast uptake signals may be due to the limited number of patients. Second, the low spatial resolution of PET results in partial volume errors that were not corrected in this study. This is especially important for LEAD since plaques are generally small in size and uptake was shown to be lower than in the carotids. FDG-PET imaging of femoral plaques is further limited by the presence of background signals in peripheral muscles. Third, even though we managed to colocalize plaque location using indicating markers throughout the whole imaging pathway as well as during atherectomy, coregistration of *in vivo* and *ex vivo* plaque remains challenging. Limitations of plaque sampling using directional atherectomy without simultaneous intravascular imaging could have contributed to the absence of the expected correlation with histopathology. Newer devices allowing simultaneous intravascular imaging during atherectomy might prove helpful for SFA plaque excision. Fourth, tissue extraction by catheter-based atherectomy allows only fractional removal of longitudinal plaque portions, which is not comparable to vessel cross-sectional imaging, impairing histologic assessment.

## Conclusion

In this study, we report on the feasibility and challenges of the simultaneous acquisition of contrast-enhanced MRI and 18-FDG PET imaging in patients with LEAD. Hybrid PET/MR imaging might yield additional value for risk estimation using more specific PET tracers. Well-designed prospective studies are needed to confirm its possible role in LEAD patients.

## Data Availability

The datasets presented in this article are not readily available because generated datasets include identification of patients enrolled and are restricted for data sharing. Requests to access the datasets should be directed to koppara@tum.de.
