# Supplementary material for: Simultaneous 18-FDG PET and MR imaging in lower extremity arterial disease
Source: Front Cardiovasc Med. 2024 Feb 9;11:1352696. doi: 10.3389/fcvm.2024.1352696 (PMC10884315; doi:10.3389/fcvm.2024.1352696)
Supplement: Supplementary file 1 [file Table1.docx]

**Supplemental Material**

| **Image Sequence** | **TOF** | **T1w TSE** | **T2w TSE** |
| --- | --- | --- | --- |
| MR Acquisition Type | 2D | 2D | 2D |
| Orientation | Tra | Tra | Tra |
| Repetition Time [ms] | 534 | 800 | 3000 |
| Echo Time [ms] | 7,2 | 12 | 73 |
| Number of Averages | 2 | 2 | 2 |
| Echo Train Length | 1 | 11 | 13 |
| PixelBandwidth [Hz / px] | 114 | 130 | 130 |
| Flip Angle [degree] | 70 | 180 | 180 |
| Pixel Spacing [mm / mm] | 0.63 / 0.63 | 0.63 / 0.63 | 0.63 / 0.63 |
| Slice Thickness [mm] | 3 | 3 | 3 |
| Matrix | 256 x 512 | 264 x 512 | 264 x 512 |
| FOV [mm x mm] | 160 x 320 | 165 x 320 | 165 x 320 |
| Acquisition Time | 7 min 33 s | 3 min 9 s | 4 min 6 s |
| Saturation | SAT1* | DB/FS** | SAT2/FS*** |

**Supplemental Table S1: MR imaging parameters**. Methods for dark blood: *saturation band to suppress venous blood flow, **dark blood (double inversion recovery = DIR), fat saturation, ***two saturation bands to suppress blood flow, fat saturation

| **Conventional AHA Classification of atherosclerotic plaque types** |
| --- |
| Type I: Initial lesion with foam cells |
| Type II: Fatty streak with multiple foam cell layers |
| Type III: Preatheroma with extracellular lipid pools |
| Type IV: Atheroma with a confluent extracellular lipid core |
| Type V: Fibroatheroma |
| Type VI: Complex plaque with possible surface defect, hemorrhage, or thrombus |
| Type VII: Calcified plaque |
| Type VIII: Fibrotic plaque without lipid core |

# Supplemental Table S2. Conventional AHA Classification of atherosclerotic plaque types

Sequence of atherosclerotic lesions from type I to type VIII.

Type I and II lesions, are combined under the term *early lesions*. Type III lesions form the bridge between early and advanced lesions. Type IV is the first lesion considered advanced by histological criteria. In this classification the term *advanced lesion* is used as a term for all lesions that disrupt intimal structure. Type V and VI lesions develop and progress by mechanisms that are superimposed on the continuing lipid accumulation that produced lesion types I through IV. In type IV lesions intimal structure is altered almost solely by an extensive accumulation of extracellular lipid localized in the deep intima (the lipid core). In type V lesions intima is thickened by substantial reparative fibrous (mainly collagenous) tissue layers (fibroatheroma). Surface defects, hematoma, and thrombotic deposits characterize type VI lesions. The predominant calcification of a fibro-lipid lesion is type VII (calcific lesion), and fibrous tissue layers without or with only minimal lipid (no core) and minimal or no calcium is type VIII (fibrotic lesion). [[22](#_ENREF_22); [23](#_ENREF_23)]
